# Comparison of the physical properties and microstructure of bigels prepared from ethyl cellulose oleogels and konjac glucomannan hydrogels containing casein or whey protein isolate

**DOI:** 10.1016/j.fochx.2025.102880

**Published:** 2025-08-05

**Authors:** Lijuan Han, Shiqi Zhang, Yingxin Chen, Lingzhi Su, Junbo He, Weinong Zhang

**Affiliations:** a Key Laboratory for Deep Processing of Major Grain and Oil, Ministry of Education, College of Food Science & Engineering, Wuhan Polytechnic University, Wuhan 430023, China; b Hubei Key Laboratory for Processing and Transformation of Agricultural Products, Wuhan Polytechnic University, Wuhan 430023, China

**Keywords:** Bigels, Binary hydrogel, Casein, Whey protein isolate

## Abstract

Bigels were prepared from ethyl cellulose oleogels and binary hydrogels containing konjac glucomannan (KGM) and two homologous proteins: casein (CS) and whey protein isolate (WPI). An increase in CS concentration led to an initial enhancement, followed by a reduction in the yield stress and hardness of the bigels. Conversely, variations in WPI concentration did not have a significant effect on these properties. As protein concentration increased, WPI-based bigels maintained the hydrogel-in-oleogel structure, whereas CS-based bigels exhibited phase inversion. The change in binary hydrogels was responsible for the structural evolution and property differences in bigels. Both CS–KGM and WPI–KGM binary hydrogels played a role in stabilizing the droplets and decreasing interfacial tension. Notably, CS–KGM mixtures demonstrated superior emulsification performance compared to WPI–KGM mixtures. By changing the type and concentrations of proteins used, different emulsification abilities can be achieved in hydrogel solutions, which in turn control the structural properties of the corresponding bigels.

## Highlights

• Proteins in binary hydrogels can alter the microstructure and properties of bigels.
• CS-based BG exhibited phase inversion as its concentration increased, while WPI-based BG did not.
• This study offers a fresh way to screen binary hydrogels for producing bigels.

## 1 Introduction

Plastic fats, owing to their semi-solid structure, impart desirable properties such as firmness and spreadability, and are widely used in food products including spreads, buttercream, sandwich biscuits, and ice cream. These products contain amounts of trans fatty acids (TFA) and saturated fatty acids (SFAs). Although partially hydrogenated oils have been largely phased out due to their high TFA content, the issue of excessive SFA intake remain a concern (Patel et al., 2020). High SFA consumption has been linked to elevated level of low-density lipoprotein cholesterol, thereby increasing the risk of cardiovascular disease (Berry et al., 2019). This has led to growing interest in developing alternatives with similar plastic functionality but reduced or no TFAs and SFAs.

Bigels (BGs) are semi-solid systems conposed of both hydrogel and organogel phases, typically prepared via high-shear mixing (Rehman & Zulfakar, 2013). They combine the functional properties of both oil- and water-based gels, often exhibiting superior performance compared to single-phase systems (Singh et al., 2014). Due to their biphasic structure, BGs exhibit enhanced mechanical strength, viscoelasticity, thermal stability, and spreadability, making them promising alternatives to traditional plastic fats in products such as margarine and spreads (Zhu et al., 2021). Importantly, BGs can be formulated to contain lower levels of SFAs, incorporate plant-based oils, and achieve desirable textural properties without the need for trans fats or fully hydrogenated oils. These characteristics make BGs attractive as clean-label and health-conscious fat substitutes in modern food formulations. Recent studies have demonstrated the successful application of BGs as fat substitutes in various food matrices, such as cookies (Quilaqueo et al., 2022) and margarine-like spreads (Steinkellner et al., 2025), highlighting their potential as health-oriented alternatives.

The hydrogel component is particularly critical in determining the texture and rheological performances of bigels. While polysaccharides such as sodium alginate, pectin, and guar gum are widely used as hydrogelators in bigels (Chao et al., 2024), protein-based hydrogels have received comparatively less attention. Most protein-based bigel research has focused on gelatin (Liu et al., 2023; Zampouni et al., 2023), followed by whey protein (Liu et al., 2024; Hashemi et al., 2023; Clímaco1 & Fasolin, 2024), whereas the use of casein (CS) remains limited. Like whey protein isolate (WPI), CS is a milk-derived protein widely employed in foods for its emulsifying, foaming, and water-binding capabilities (Li et al., 2021; Ranadheera et al., 2016). These functional attributes make CS a promising, yet underutilized, candidate for structuring bigels. Although Pickering bigels stabilized by protein microgels have recently been explored to enhance digestion and oral properties (Liu et al., 2024), limited work has addressed systems utilizing molecular emulsifiers such as protein-polysaccharide complexes. Such systems may offer improved tunability of interfacial behavior and gel structure, expanding the design flexibility of food-grade bigels.

In this context, binary hydrogels composed of protein–polysaccharide combinations have gained attention due to their versatile structural and mechanical properties (Liu et al., 2019a; Wang et al., 2021). Systems like gelatin–konjac gum and gelatin–κ-carrageenan have demonstrated promising potential in bigel applications (Liu et al., 2023; Zampouni et al., 2023). However, combinations involving CS or WPI with konjac glucomannan (KGM) remain largely unexplored, despite the possibility of forming structured hydrogels with tailored rheological and emulsifying characteristics.

Building on this gap, the present study aims to systematically compare the effects of CS and WPI in KGM-based binary hydrogels, incorporated into bigels structured with ethyl cellulose organogels. Two homologous milk proteins with distinct molecular weights and interfacial properties were selected to investigate how protein type and concentration influence the structure, stability, and mechanical behavior of bigels. To this end, comparative analyses of interfacial parameters were conducted, including contact angle, dynamic interfacial tension, hydrophilic–lipophilic balance (HLB), intrinsic fluorescence, and surface hydrophobicity. These characteristics were further correlated with hydrogel behavior and the resulting bigel structure. This approach provides new insights into the role of protein composition in designing functional, plant-oil-based bigels with potential applications in spreadable fat alternatives.

## 2 Materials and methods

### 2.1 Materials

Ethyl cellulose (EC) with a viscosity of 9–11 mPa·s at a concentration of 5 % in a mixture of toluene and ethanol (80:20 *v*/v) and konjac glucomannan (KGM) with a viscosity greater than 15,000 mPa·s, were acquired from Aladdin Inc. in Shanghai, China. Food-grade casein (CS) and whey protein isolate (WPI) powder were acquired from Shanghai Yuanye Biotechnology Co., Ltd. (Shanghai, China). Rice bran oil (RBO, food grade) was obtained from Yihai Kerry Food Co., Ltd. (Shanghai, China). Deionized water was utilized in all of the conducted studies. Nile red and Nile blue (>95 % purity) were obtained from Aladdin Inc. in Shanghai, China. Liquid Paraffin were purchased from Ke Shi Co., Ltd. Turpentine was acquired from Shanghai Macklin Biochemical Co., Ltd. (Shanghai, China). All chemicals were of analytical grade and used as received.

### 2.2 Bigel preparation

Oleogels and hydrogels were prepared according to our previously reported method (Su et al., 2024). EC (6 wt%) was dissolved in RBO at 150 °C for 15 min under stirring (200 rpm), then cooled in a 60 °C water bath to form oleogels. A protein–polysaccharide binary mixture was prepared by dissolving CS or WPI (0.1, 1, 2, or 4 wt%) and 0.5 wt% KGM in deionized water, followed by heating at 85 °C for 25 min and storage at 4 °C for 24 h. These binary mixtures were labeled as 0.1CS–KGM, 1CS–KGM, 2CS–KGM, 4CS–KGM, and 0.1WPI–KGM, 1WPI–KGM, 2WPI–KGM, 4WPI–KGM, respectively.

Bigels were obtained by mixing the oleogel to the aqueous solution at a 60:40 (*w*/w) ratio at 60 °C with magnetic stirring (200 rpm, 10 min), followed by high-speed homogenization (12,000 rpm, 3 min; T18, IKA, Germany). The resulting bigels were stored for 24 h at 4 °C before analysis and designated as BG (0.1 % CS or WPI), BG (1 % CS or WPI), BG (2 % CS or WPI), and BG (4 % CS or WPI), respectively. Unless specified otherwise, all binary mixtures and BGs contained 0.5 % KGM.

### 2.3 Characterization of bigels

#### 2.3.1 Texture analysis of bigels

The texture analyzer (TMS-Pro, FTC, USA) was used to measure the hardness and gumminess of bigels. To perform the measurement, a plane probe with a 25 mm diameter (P/25) was equipped. The samples loaded in beakers were subjected to the single extrusion model with pre-testing, testing, and post-testing rates of 5 mm/s, 1 mm/s, and 5 mm/s, respectively. For the measurement, the trigger force was set at 0.05 N, and the strain was set at 50 %.

#### 2.3.2 Rheological measurement of bigels

The rheological study was performed under a Kinexus Pro+ rheometer (Malvern, UK) equipped with a parallel plate geometry (40 mm in diameter, 1 mm gap). Amplitude sweep test was carried out under conditions of 1 Hz frequency at 25 °C to determine the linear viscoelastic region, and the strain was varied from 0. 01 % to 100 %. The frequency sweep was performed with a 0.1 % strain at 25 °C as the frequency was varied between 0.1 and 100 Hz. The yield stress (σy) was determined by conducting measurements in triplicate at 25 °C, which was defined as the stress value where the elastic modulus G′ shows a 5 % decrease (Wang et al., 2024).

#### 2.3.3 Microscopical observations of bigels

The microstructure and droplet size distribution of BGs was investigated using a confocal laser scanning microscope (CLSM; FV1200-OSR, Olympus Corporation, Japan). Nile red (0.1 wt%) and Nile blue (0.1 wt%) were added to the oil and aqueous phases, respectively, at a volume-to-weight ratio of 1:40 (Vershkov & Davidovich-Pinhas, 2023) to stain the oil phase red (excitation: 543 nm) and the proteins (CS or WPI) blue (excitation: 610 nm). This dyeing approach enhanced droplet contrast and image clarity. Mean droplet size was calculated by randomly measuring at least 200 droplets using Image-J software v1.53 (NIH, USA).

The network structure and interfacial features of BGs were further observed by cryo-SEM (Quanta 450, FEI, USA). Samples were sublimated at −90 °C for 10 min, then sputter-coated with gold (10 mA, 60 s). Imaging was performed at −140 °C with an accelerating voltage of 5 kv (Guo et al., 2023).

#### 2.3.4 The mobility of hydrogen protons measurements of CS or WPI bigels

For the analysis of LF-NMR relaxation measurements, a NMI20-040 V-I spectrometer (Niumag Electric Corporation, China) was utilized and operated at a resonance frequency of 21 MHz. Approximately 10 g sample was placed in a 25 mm NMR tube covered with a Teflon film. Carr-Purcell-Meiboom-Gill (CPMG) pulse sequence was employed for conducting transverse relaxation time (*T*_2_) measurements at 32 °C. The pulse sequence parameters included a wait time of 2000 ms, an echo time of 0.080 ms, 5000 echoes, and 4 repeated scans. The 90°and 180°pulse widths were set to 8 μs and 16 μs, respectively. Subsequently, the CPMG decay curves obtained were analyzed using the instrument's built-in MultiExp Inverse Laplace Transformation software (exact version not disclosed by the manufacturer). A Tikhonov regularization approach with non-negative constraint was applied, consistent with commonly used algorithms in similar LF-NMR studies (Zhong et al., 2022). The analysis involved calculations of the intrinsic *T*_2_ peak positions, peak area (A_2_), and the relative percentage of each peak (%). These measurements were carried out in triplicate.

### 2.4 Characterization of the CS–KGM or WPI–KGM binary mixtures

#### 2.4.1 Measurement of contact angle of the CS–KGM or WPI–KGM binary mixtures

Contact angles were measured on a contact angle meter and recorded with ADVANCE software according to the published method of Lee et al. (2019) with some appropriate modifications. The contact angles of CS–KGM or WPI–KGM aqueous solutions (0.1–4 wt% protein, 0.5 % KGM) on the surface of ethyl cellulose oleogel films were measured.

#### 2.4.2 Dynamic interfacial tension of the CS-KGM or WPI-KGM binary mixtures

The adsorption kinetics of the binary mixtures at the oil/water interface were assessed using dynamic interface tension. It was determined by a drop shape analyzer (DSA25, Krüss, Germany). CS–KGM or WPI–KGM aqueous solutions (0.1–4 wt% protein, 0.5 % KGM) and rice bran oil were put in the high-precision syringe and cuvette, respectively. The test was conducted using the pendant drop method with a volume of 30 μL, and measurements were taken 3600 times at a temperature of 25 °C (Hou & Xu, 2023).

#### 2.4.3 The range of HLB values of the CS-KGM or WPI-KGM binary mixtures

Liquid paraffin and turpentine oil were blended to prepare oil phases with varying Hydrophilic Lipophilic Balance (HLB) values (2, 4, 6, 8, 10, 12). These oil phases were mixed with CS–KGM or WPI–KGM aqueous solutions (0.1–4 wt% protein, 0.5 wt% KGM), and the resulting emulsion stratification was observed. This methodology was designed to determine the variation in HLB value ranges of the hydrogel solutions, thus evaluating their contribution to the bigels formation process (Jiang et al., 2022; Wang et al., 2023). The experiments were conducted at 60 °C, maintaining the temperature conditions consistent with those used in bigels production.

#### 2.4.4 Surface hydrophobicity of the CS-KGM or WPI-KGM binary mixtures

The surface hydrophobicity of the binary mixtures (CS-KGM or WPI-KGM) was investigated according to Liu et al. (2019b) with 8-anilino-1-naphthalenesulfonic acid (ANS). The fluorescence intensity of the binary mixture suspension (4 mL) with concentration ranging from 0.05 mg/mL to 0.5 mg/mL was determined using a fluorescence spectrophotometer (F-4600, HI-TACHI, Japan). For the analysis, 20 μL of ANS solution (8 mM in 0.01 M phosphate buffer, pH 7.0) was added to the suspension. Fluorescence intensity was recorded using a spectrophotometer (F-4600, HITACHI, Japan) at excitation/emission wavelengths of 390/470 nm, with both slits set to 5 nm. The slope (H_0_) of the linear plot showing relative fluorescence intensity versus protein concentration served as the indicator of the binary mixtures surface hydrophobicity. The linear regression fitting and slope calculation were performed using Origin software (version 2021, Origin Lab Corporation, USA).

#### 2.4.5 Intrinsic fluorescence analysis of the CS-KGM or WPI-KGM binary mixtures

The intrinsic fluorescence emission spectra of heat-treated protein without KGM (1CS–0KGM, 1WPI–0KGM) and protein–KGM binary mixtures (1CS–0.2KGM, 1CS–KGM, 1WPI–0.2KGM, 1WPI–0.2KGM, 1WPI–KGM) were measured at 25 °C using a fluorescence spectrophotometer (F-4600, Hitachi, Japan) according to the method (Wang et al., 2019) with some modifications. The samples were centrifuged at 5000 ×*g* for 10 min to collect the supernatants. The protein concentration in the supernatants was determined and then diluted to 0.15 mg/mL. Excitation wavelength was set at 290 nm, and the emission spectra were measured between 300 nm and 400 nm, with both excitation and emission slits set to 5 nm. Fluorescence data processing and spectral analysis were performed using FL Solutions software (version 4.0, Hitachi High-Tech Corporation, Japan).

### 2.5 Statistical analysis

The results obtained from the experiments, which were carried out at least in triplicate unless stated otherwise, were subjected to analysis using Origin 2021 software (Origin Lab, USA). The data was presented as mean ± standard deviation. Initial analysis involved conducting a one-way analysis of variance (ANOVA), which was followed by Duncan's test, aiming to determine significant differences (*P* < 0.05).

## 3 Results and discussion

### 3.1 Mechanical and rheological properties of bigels with varying concentrations of CS and WPI

The TPA test was used to evaluate the textural properties of bigels with varying concentrations of CS and WPI. Table 1 displayed the results obtained from the analysis. Among the samples tested, the BG (1 % CS) sample exhibited the highest levels of hardness, gumminess, and chewiness. Comparatively, the cohesiveness of the WPI group bigels surpassed that of the CS group bigels significantly. Notably, increasing the CS concentration led to significant changes in the hardness, gumminess, and chewiness of the bigels samples. Conversely, variations in WPI concentration did not yield significant differences in the data of bigels samples. This discrepancy could be attributed to the inherent characteristics and properties of the proteins involved. Similar observations have been reported by Liu et al. (2024), who found that the type and concentration of proteins significantly influence the texture and oral properties of bigels. The findings suggest that the protein type within the hydrogel matrix plays a crucial role in modulating the textural properties of bigels, indicating the potential for tailored design based on protein selection within the bigels system.

**Table 1:** Textural properties of bigels with different CS or WPI concentrations.

Sample	Hardness (N)	Cohesiveness	Springiness (mm)	Gumminess (N)	Chewiness (mJ)
BG (0.1 %CS)	0.104 ± 0.001^b^	0.596 ± 0.005^b^	7.136 ± 0.351^d^	0.063 ± 0.001^c^	0.445 ± 0.025^c^
BG (1 %CS)	0.124 ± 0.002^a^	0.590 ± 0.008^b^	7.936 ± 0.493^cd^	0.076 ± 0.001^a^	0.767 ± 0.114^a^
BG (2 %CS)	0.059 ± 0.007^d^	0.602 ± 0.035^b^	2.443 ± 0.377^e^	0.037 ± 0.005^d^	0.069 ± 0.003^d^
BG (4 %CS)	0.108 ± 0.008^b^	0.605 ± 0.009^b^	8.995 ± 0.600^b^	0.065 ± 0.006^bc^	0.557 ± 0.016^bc^
BG (0.1 %WPI)	0.105 ± 0.004^b^	0.712 ± 0.040^a^	8.495 ± 0.642^bc^	0.075 ± 0.002^a^	0.594 ± 0.0120^b^
BG (1 %WPI)	0.089 ± 0.001^c^	0.751 ± 0.008^a^	8.725 ± 0.894^bc^	0.068 ± 0.001^b^	0.606 ± 0.055^b^
BG (2 %WPI)	0.106 ± 0.008^b^	0.715 ± 0.017^a^	9.895 ± 0.153^a^	0.077 ± 0.001^a^	0.803 ± 0.132^a^
BG (4 %WPI)	0.103 ± 0.006^b^	0.716 ± 0.027^a^	10.461 ± 0.202^a^	0.073 ± 0.002^a^	0.775 ± 0.054^a^

The values expressed represent the mean ± SD. ^a-d^: Different letters represent significant differences (*P* < 0.05).

**Fig. 1:**
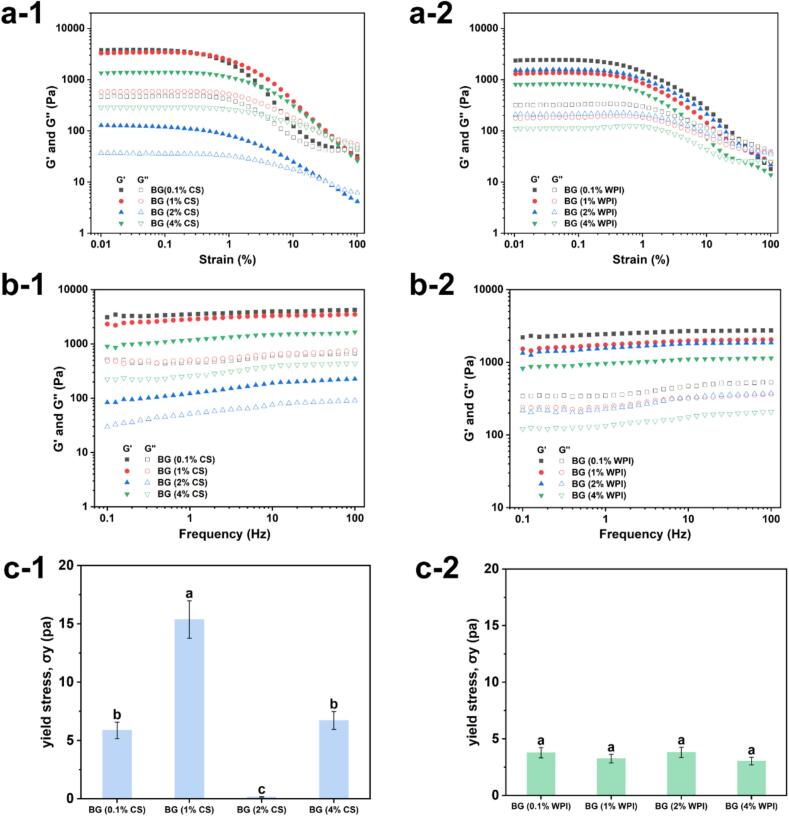
The storage modulus (G′) and loss modulus (G″) of bigels prepared with varying concentrations (0.1 %, 1 %, 2 %, 4 %) of CS or WPI during (a) strain sweep and (b) frequency sweep tests. (c) Yield stress (σy) of the corresponding bigels.

Rheological testing was conducted to characterize the rheological properties of bigels, as illustrated in Fig. 1. In the strain sweep tests (Fig. 1a), all bigels exhibited a typical gel-like behavior in the linear viscoelastic region, where the storage modulus (G′) was higher than the loss modulus (G″). As strain increased, a crossover point appeared where G″ surpassed G′, indicating the yield point and the onset of structural breakdown of the gel network. It is noteworthy that BG (2 % CS) exhibited the lowest G′ value, suggesting its weaker solid-like characteristics compared to the other bigels. Moreover, as the CS or WPI concentration in the hydrogel increased, both the storage modulus and the loss modulus exhibited a declining trend. The gel-like nature of the bigels was further validated by the continuous frequency sweep spanning from 0.1 to 100 Hz, where the G′ values significantly exceeded the G″ values. The frequency-dependent behavior was also evident from the slight frequency dependency observed in both G′ and G″ across all samples, as depicted in Fig. 1b. This further supports the existence of a structured network under small deformations, although the strain sweep results indicate that this network is susceptible to disruption under higher strain amplitudes.

**Fig. 2:**
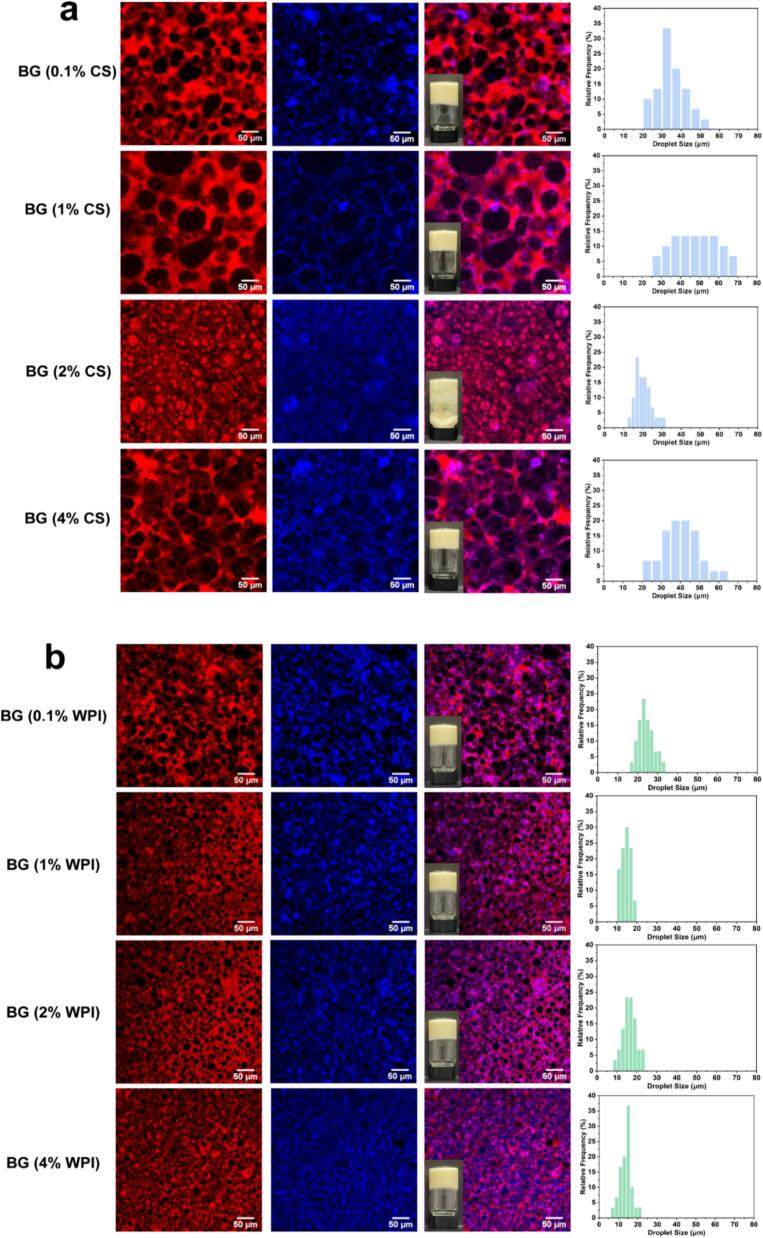

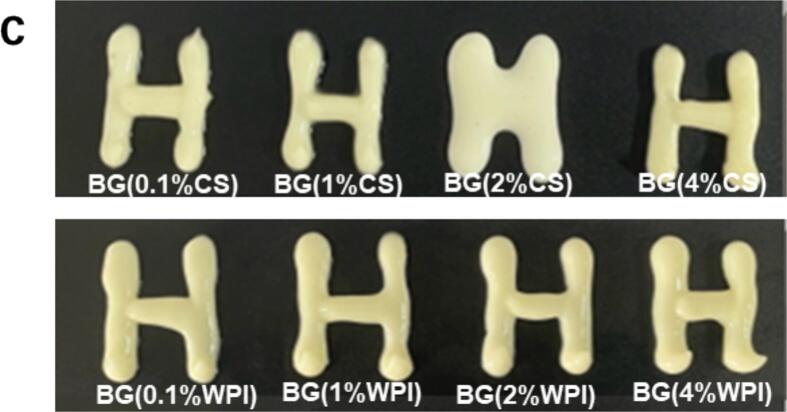
Micrographs and droplet distribution images of bigels containing different concentrations of (a) CS and (b) WPI in the hydrogel were captured using CLSM. The oil phase is stained red (Nile Red) and the protein–KGM phase is blue (Nile Blue). Scale bar = 50 μm. And (c) visual appearances of bigels (with varied CS or WPI concentrations) used as margarine. (For interpretation of the references to colour in this figure legend, the reader is referred to the web version of this article.)

The yield stress values (σy) depicted in Fig. 1c provided a clearer insight into the impact of CS and WPI on the rheological properties of the bigels. These values corresponded to the mechanical characteristics of the bigels using the 95 % criterion in the linear viscoelastic region response. In Fig. 1c-1, the yield stress values of bigels exhibited a notable increase followed by a decrease, with a higher yield stress indicating enhanced material stability when subjected to external forces, thereby reducing the propensity for flow or deformation (Liu et al., 2019c). Notably, the highest yield stress (15.37 Pa) was observed for BG (1 %CS) (cf. ∼9 Pa at 0.1 % CS and ∼ 10.8 Pa at 4 % CS), indicating that the BG (1 %CS) has the strongest network and greatest mechanical stability under stress. On the other hand, Fig. 1c-2 illustrated that the yield stress values remained relatively constant as the WPI concentration increased, indicating a limited impact of WPI on the rheological properties of the bigels.

### 3.2 Microstructure of bigels with varying concentrations of CS and WPI

Fig. 2 displays the CLSM, droplet size distribution images, and visual appearances of bigels prepared with different CS or WPI concentrations. Bigels prepared using CS-KGM exhibited a phase inversion phenomenon, with BG (2 %CS) showcasing an O/W structure while the other samples displayed a W/O structure. All bigel samples were macroscopically homogeneous and self-standing after preparation, with no observable phase separation, confirming that all the bigels were immediately stable. Notably, the BG (2 %CS) sample flowed when inverted and exhibited an unclear surface texture compared with the other bigels (Fig. 2c). A noteworthy observation was the increase in droplet diameter and the closer packing of droplets in the W/O structure with the rise in CS concentration (Fig. 2a). However, this trend was not strictly monotonic. While BG (0.1 %CS) had a mean droplet diameter of 8.2 ± 0.5 μm and BG (4 %CS) reached 18.4 ± 0.6 μm, BG (2 %CS) displayed a notebly smaller droplet size despite its intermediate concentration. This anomaly can be attributed to the phase inversion observed in BG (2 %CS), where the system transitioned from a water-in-oil (W/O) to an oil-in-water (O/W) structure, as confirmed by CLSM and cyro-SEM images. In the O/W structure, oil droplets are more uniformly dispersed in the continuous hydrogel matrix, resulting in smaller and more stable droplets. Such inversion significantly alters the emulsification behavior, droplet size distribution, and gel morphology. Beyond structural inversion, the protein concentration also affects interfacial adsorption and matrix viscosity, which may further contribute to the observed size variation. Conversely, bigels prepared using WPI-KGM all exhibited a W/O structure as shown in Fig. 2b, and all samples were self-standing when inverted. In the WPI series, droplet diameter gradually decreased from 12.7 ± 0.5 μm (BG (0.1 %WPI)) to 6.9 ± 0.3 μm (BG (4 %WPI)) as the WPI concentration increased and became more uniformly spherical. These distinctions in droplet structures and behaviors are attributable to the unique properties of CS and WPI, which dictate their emulsifying capabilities and ultimately impact the characteristics of the resulting bigels. Bigels are attracting interest as healthier fat replacers in foods. Quilaqueo et al. (2022) demonstrated bigels as replacements for saturated fat in cookies, and Steinkellner et al. (2025) developed bigel-based margarine with favorable consistency. The visual appearances of bigels (Fig. 2c) were reminiscent of margarine, indicating its potential as a low-saturated-fat margarine or spread. Although we did not conduct sensory tests, these analogies suggest promising applications in bakery and spread products.

**Fig. 3:**
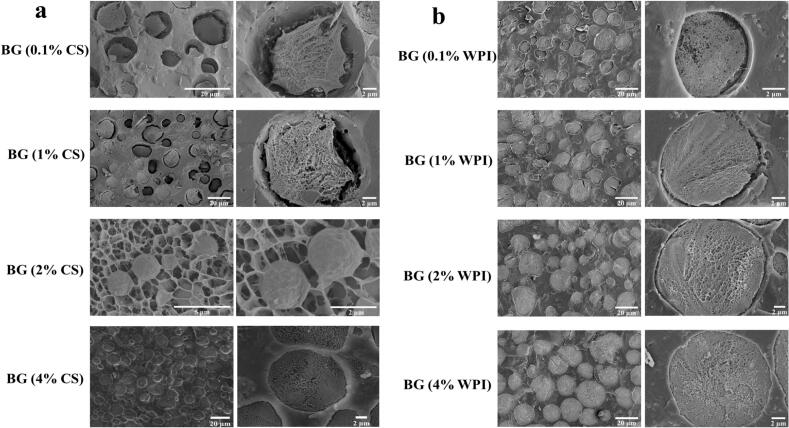
Cryo-SEM micrographs of bigels (BG) with different protein–KGM concentrations: (a) casein (CS) series—BG (0.1 %CS), BG (1 %CS), BG (2 % CS), BG (4 %CS); (b) whey protein isolate (WPI) series—BG (0.1 %WPI), BG (1 %WPI), BG (2 %WPI), BG (4 %WPI).

Through cryo-SEM analysis, the microstructural variances within bigels were further explored by examining four BG samples with differing protein concentrations to elucidate the microstructure of bigels with varying CS or WPI concentrations. According to the findings presented in Fig. 3, it could be deduced that the network configuration inside the dispersed water phase droplets in the W/O type sample illustrated in Fig. 3 was established by CS–KGM or WPI–KGM. Specifically, in the BG (0.1 %CS) and BG (1 %CS) specimens depicted in Fig. 3a, the KGM-induced network constrained the migration of CS, resulting in the absence of conspicuous CS distribution at the oil-water interface and instigating condensation deformation within the internal network. In contrast, BG (2 %CS) exhibited an O/W-type organization, with oil droplets ensconced within the CS–KGM network. As shown in Fig. 3b, with an increase in WPI concentration, the voids at the droplet oil-water interface were gradually filled, and no notable condensation deformation was discernible within the internal network.

From the microscopic observations mentioned earlier, it is clear that there are notable structural disparities in the bigels made with two distinct proteins, CS and WPI. These differences can be attributed to the inherent properties of the two proteins, particularly their molecular weights and interfacial behavior. SDS-PAGE analysis (Fig. S1) revealed that CS exhibited major protein bands in the 35–50 kDa range and above, while WPI bands were mainly concentrated around 25–30 kDa. The higher molecular weight and aggregation tendency of CS may enhance its ability to form a more robust interfacial film around oil droplets. This likely accounts for the superior emulsifying performance and structural stability observed in CS-based bigels, while WPI, with its smaller molecular size, contributes less to the formation of continuous gel networks.

Beyond interfacial stabilization, these compositional differences also appear to influence the phase behavior of the bigel systems. Phase inversion in CS-based bigels at higher protein concentrations may be attributed to the increased hydrophobicity and micellar size of CS, which promote oil droplet encapsulation within a continuous aqueous network. A similar inversion phenomenon has been reported by Zampouni et al. (2023), where increasing the gelatin content in a κ-carrageenan hydrogel system led to a shift from W/O to O/W-type bigels, supporting the role of protein-induced matrix dominance in structural transitions. Our results align with these mechanisms and with the literature on bigel properties (Chao et al., 2024).

### 3.3 LF-NMR relaxation behavior of bigels with varying concentrations of CS and WPI

**Fig. 4:**
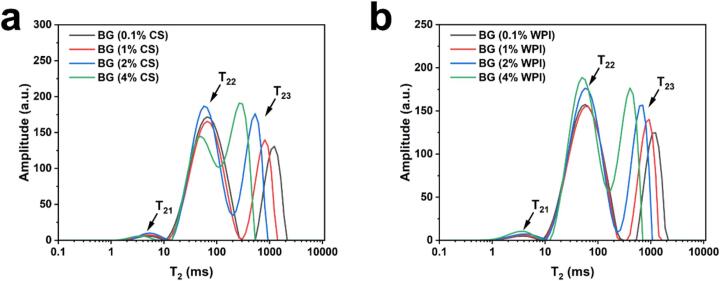
Transverse relaxation properties of bigels (BG) with different protein–KGM concentrations: (a) casein (CS) series—BG (0.1 %CS), BG (1 %CS), BG (2 %CS), BG (4 %CS); (b) whey protein isolate (WPI) series—BG (0.1 %WPI), BG (1 %WPI), BG (2 %WPI), BG (4 %WPI). Distribution of the transverse relaxation time (*T*_2_) spectra.

As shown in Fig. 4, the *T*_2_ relaxation profiles of BG samples containing different concentrations of CS or WPI exhibited three distinct peaks, corresponding to *T*_21_, *T*_22_, and *T*_23_. Remarkably, as the concentration of CS or WPI increased, the *T*_21_ and *T*_22_ peaks remained relatively stable, while the *T*_23_ peak underwent a noticeable leftward shift. This shift toward the left signifies a reduction in the *T*_2_ relaxation time, suggesting a heightened constraint on the hydrogen protons that are more mobile within the double gel system. This phenomenon could be attributed to the escalating concentration of CS or WPI in the aqueous phase, enhancing the binding of CS/WPI with water molecules, consequently impeding the mobility of the water molecules (Zhong et al., 2022). As shown in Table 2, at the same protein concentration, the A_23_ (%) in the CS group samples significantly surpassed that of the WPI group samples (*p* < 0.05), whereas the A_22_ (%) was notably lower in the CS group compared to the WPI group. This variation implies that CS induces a greater conversion of immobile water into free water, potentially compromising the stability of the bigels system.

**Table 2:** Peak time(*T*_2_), peak areas(A_2_) and fractions of peak areas (%) of bigels.

Sample	*T*_2_ peak time (ms)			A_2_ peak area			Fractions of peak areas (%)		
	T_21_	T_22_	T_23_	A_21_	A_22_	A_23_	A_21_	A_22_	A_23_
BG (0.1 % CS)	4.64 ± 0.0^b^	65.79 ± 0.0^a^	1232.85 ± 0.0^a^	40.05 ± 2.0^d^	2180.80 ± 10^a^	763.53 ± 5.0^f^	1.34 ± 0.06^f^	73.07 ± 0.09^a^	25.58 ± 0.11^g^
BG (1 % CS)	5.34 ± 0.0^a^	65.79 ± 0.0^a^	811.13 ± 0.0^c^	57.16 ± 6.0^c^	1947.39 ± 7.0^e^	813.17 ± 3.0^e^	2.03 ± 0.21^cd^	69.11 ± 0.21^c^	28.86 ± 0.04^e^
BG (2 % CS)	5.34 ± 0.0^a^	57.22 ± 0.0^cd^	533.67 ± 0.0^e^	73.95 ± 1.0^b^	1986.76 ± 12^d^	1116.21 ± 3.0^c^	2.33 ± 0.04^bc^	62.54 ± 0.20^e^	35.14 ± 0.16^b^
BG (4 % CS)	3.36 ± 0.26^d^	49.77 ± 0.0^e^	265.61 ± 0.0^g^	49.95 ± 6.0^c^	1515.77 ± 19^f^	1449.71 ± 9.0^a^	1.66 ± 0.21^ef^	50.27 ± 0.44^g^	48.08 ± 0.49^a^
BG (0.1 % WPI)	3.38 ± 0.57^d^	57.22 ± 0.0^cd^	1232.85 ± 0.0^a^	51.33 ± 11.0^c^	2040.46 ± 9.0^b^	707.89 ± 7.0^g^	1.83 ± 0.38^de^	72.88 ± 0.18^a^	25.28 ± 0.20^g^
BG (1 % WPI)	3.86 ± 0.30^cd^	62.94 ± 4.95^ab^	932.6 ± 0.0^b^	57.80 ± 5.0^c^	1937.96 ± 10^e^	776.83 ± 8.0^f^	2.09 ± 0.20^cd^	69.9 ± 0.07^b^	28.02 ± 0.15^f^
BG (2 % WPI)	4.24 ± 0.35^bc^	60.08 ± 4.95^bc^	705.48 ± 0.0^d^	74.70 ± 3.0^b^	2053.73 ± 13^b^	929.7 ± 4.0^d^	2.44 ± 0.10^b^	67.16 ± 0.08^d^	30.4 ± 0.08^d^
BG (4 % WPI)	3.51 ± 0.0^d^	52.25 ± 4.30^de^	403.7 ± 0.0^f^	94.32 ± 2.0^a^	2012.3 ± 26^c^	1183.82 ± 34^b^	2.87 ± 0.05^a^	61.16 ± 0.94^f^	35.98 ± 0.95^c^

Letters of different lines indicate significantly different (*P* < 0.05).

### 3.4 Characterization of the CS–KGM and WPI–KGM binary mixtures

The differences in rheological properties, texture performance, and microstructure between CS-based or WPI-based BGs primarily caused by the distinct properties of their binary mixtures (CS–KGM or WPI–KGM) in aqueous-phase. Therefore, a comprehensive study of these binary mixtures is critical and necessary.

#### 3.4.1 Contact angle and interfacial dilational propertie of the binary mixtures

**Fig. 5:**
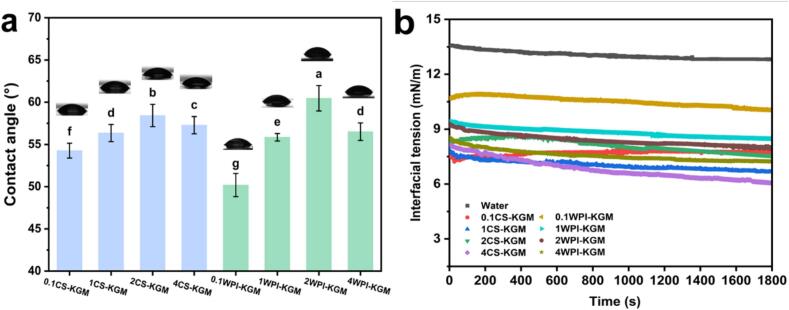
(a) The contact angles of different CS–KGM or WPI–KGM binary mixtures (0.1–4 % protein, 0.5 % KGM) on the surface of ethyl cellulose oleogel films. (b) Dynamic interface tension of CS-KGM or WPI-KGM binary mixtures (0.1–4 % protein, 0.5 % KGM). Different superscript letters (a-g) in the figure indicated significant difference (*P* < 0.05).

The interfacial properties and wetting behavior between a liquid and a solid surface are commonly characterized by the contact angle (Shi et al., 2023). As illustrated in Fig. 5a, all samples exhibited contact angles below 90° on the EC oleogel surface, indicating favorable wettability and interfacial compatibility between the oleogel and the CS–KGM or WPI–KGM binary mixtures. With increasing CS or WPI concentration, the contact angle initially increased and then decreased in both groups, with the highest values observed at 2 % protein concentration. This trend may be attributed to the similar amphiphilic nature of CS and WPI, both derived from milk proteins, which affects interfacial adsorption and droplet structure. A larger contact angle implies reduced spreading of the binary mixture droplet on the oleogel surface, suggesting stronger droplet cohesion and potentially enhanced internal structural stability.

The dynamic interfacial tension curves of CS–KGM or WPI–KGM binary mixtures at the oil–water interface are presented in Fig. 5b. Interfacial tension is closely associated with emulsion formation and stability (Amine et al., 2014) and reflects the interfacial adsorption dynamics of emulsifying agents. In all samples, the interface tension began at a moderately low level and gradually reached equilibrium, showing a typical time-dependent decrease. This behavior suggests that the adsorption of protein–polysaccharide mixtures to the interface occurred relatively slowly. The absence of a sharp decline may be attributed to the KGM matrix, which likely restricted the diffusion of CS or WPI to the interface, thereby slowing the adsorption process and resulting in a smoother tension curve. Since both CS and WPI exhibited surface activity, they could reduce the interfacial tension between oil and water. However, the interfacial tension of 0.1WPI–KGM was higher than that of the other samples, which may be attributed to the lower molecular weight of WPI and its limited ability to form stable interfacial networks. As confirmed by SDS-PAGE analysis (Fig. S1), CS exhibits larger molecular weight bands than WPI, likely contributing to the superior emulsifying capacity and lower interfacial tension of CS–KGM mixtures.

#### 3.4.2 Analysis of HLB value range of CS-KGM and WPI-KGM binary mixtures

**Fig. 6:**
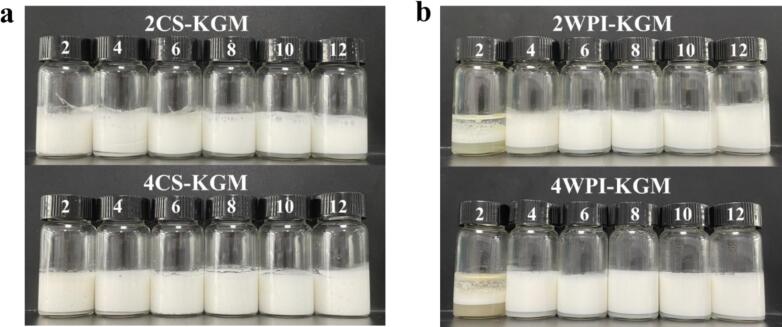
Images of emulsion morphology of (a) CS–KGM and (b) WPI–KGM binary mixtures (2 % and 4 % protein, 0.5 % KGM) in oil phases with various HLB values (2, 4, 6, 8, 10, 12).

Wang et al. (2023) demonstrated that optimal emulsion stability is achieved when the HLB value of the emulsifying system closely matches that of the oil phase. To examine differences in emulsification behavior between two milk proteins of differing molecular weights, we determined the effective HLB range of selected hydrogel solutions. Given the relatively slow interfacial adsorption kinetics and similar interfacial rheological profiles of WPI and CS, samples with higher protein concentrations (2 % and 4 %) were selected for this analysis. As illustrated in Fig. 6a, 2CS–KGM and 4CS–KGM exhibited effective emulsification across oils with various HLB values. In contrast, 2WPI–KGM and 4WPI–KGM displayed poor emulsifying performance in low-HLB oil (HLB = 2), leading to noticeable phase separation (Fig. 6b). The superior emulsifying capacity of CS–KGM compared to WPI–KGM may be attributed to its higher surface hydrophobicity, which facilitates greater protein adsorption at the oil–water interface and the formation of a more cohesive interfacial film. These findings indicates that the CS–KGM system has broader HLB compatibility than the WPI–KGM counterpart. Therefore, by manipulating protein type and concentration, hydrogel emulsifiers with distinct emulsification ranges can be designed to meet the needs of diverse oil phases.

In addition, KGM is known to interact with proteins at the oil-water interface, enhancing droplet stabilization through the formation of a viscous interfacial network. Bi et al. (2025) reported that KGM–WPI complexes contributed to the stability and uniformity of multiple emulsions. In our system, a similar mechanism likely occurs, where KGM enhances the interfacial network formation and improves the overall structural stability of both CS- and WPI-based bigels.

#### 3.4.3 Surface hydrophobicity and intrinsic fluorescence analysis of the binary mixtures

**Fig. 7:**
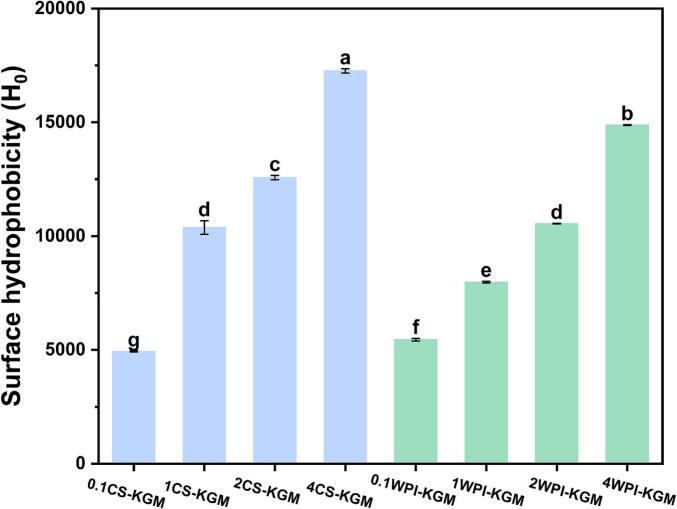
Surface hydrophobicity (H_0_) of (a) CS–KGM and (b) WPI–KGM binary mixtures (0.1–4 % protein, 0.5 % KGM). Different superscript letters (a-g) in the figure indicated significant difference (*P* < 0.05).

Surface hydrophobicity is used to assess the conformational changes of a protein by indicating the quantity of exposed hydrophobic groups on its surface. In the natural state, polar molecules are typically found on the protein surface, while nonpolar molecules are typically embedded internally as hydrophobic cores (Sheng et al., 2017). As shown in Fig. 7, with the increase in CS or WPI concentration in the protein–KGM mixture, the surface hydrophobicity of both groups of samples significantly increased (*P* < 0.05), and the surface hydrophobicity of the CS group samples was higher than that of the WPI group. This could be due to the higher content of hydrophobic amino acids in CS, resulting in most of the hydrophobic residues on the molecule being exposed on its surface, thereby exhibiting higher surface hydrophobicity (Nwachukwu & Aluko, 2018). The increase in surface hydrophobicity is associated with an improvement in emulsification effects.

These interfacial differences between CS–KGM and WPI–KGM systems reflect distinct emulsifying capacities, which can be attributed to differences in protein molecular weight, hydrophobicity, and network-forming ability. Compared with our molecularly emulsified bigels, Liu et al. (2024) reported a structurally different class of Pickering bigels stabilized by whey protein microgels. In their system, the enhanced interfacial stability was primarily achieved through the irreversible adsorption of colloidal protein particles at the oil–water interface, forming a rigid protective shell around droplets. This particle-based mechanism differs from our approach, where interfacial stabilization relies on molecular adsorption and the formation of protein–KGM complexes that reduce interfacial tension and strengthen the continuous phase.

While both strategies aim to improve emulsion stability, our CS–KGM system demonstrated lower interfacial tension and stronger hydrophobic interactions than the WPI–KGM system, resulting in better emulsification performance at the molecular level. These findings suggest that protein-polysaccharide binary mixtures offer a tunable alternative to particle-stabilized bigels, especially for applications requiring precise modulation of interface properties and gel structure.

**Fig. 8:**
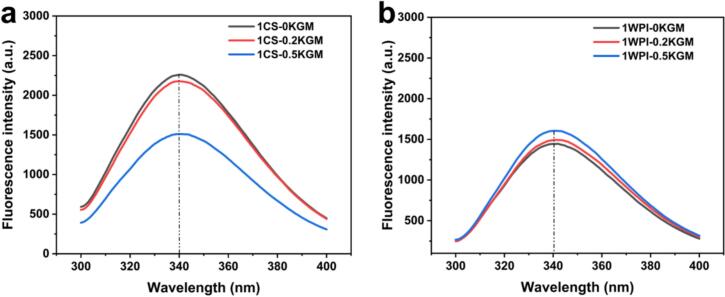
Intrinsic fluorescence emission spectra of (a) CS–KGM and (b) WPI–KGM binary mixtures with a fixed protein concentration (1 %) and increasing KGM concentrations (0, 0.2, and 0.5 %).

In order to examine the impact of KGM concentration on the proteins, the conformational differences at the tertiary structure level were analyzed using intrinsic fluorescence emission spectra in samples with KGM concentrations of 0 %, 0.2 %, and 0.5 % under a fixed CS or WPI concentration (1 %) in the protein-polysaccharide mixture. Fig. 8 illustrates that samples with different KGM concentrations did not show red or blue shifts, indicating only a variation in fluorescence intensity. In the CS group samples, an increase in KGM concentration led to a decrease in fluorescence intensity. This reduction can be attributed to complex formation between tryptophan in the protein and KGM, which reduces the number of tryptophan capable of emitting fluorescence, thereby inducing a fluorescence quenching effect (Fig. 8a). This quenching behavior has also been reported in other protein-polysaccharide systems and is generally attributed to conformational changes or shielding of fluorophores caused by complexation (Song et al., 2023). Conversely, the fluorescence intensity of the WPI group samples increased as the KGM concentration rose, potentially because of alterations in the solubility or aggregation state of WPI induced by KGM addition, indirectly influencing the fluorescence intensity (Fig. 8b).

### 3.5 Overall comparison of CS- and WPI-based bigels and application perspective

These findings suggests that CS-based bigels possess favorble attributes for potential food applications. In particular, their enhanced gel strength, structural integrity, and margarine-like appearance (Fig. 3c) point to their suitability as alternatives to traditional plasticized fats in products such as spreads and bakery fillings. Unlike conventional fat systems that rely on high levels of saturated fatty acids, the CS-based bigels in this study exhibit reduced free oil content and improved mechanical stability, offering a promising route toward cleaner-label, health-oriented fat replacements. Future studies should evaluate the sensory attributes and thermal performance of these bigels in real food matrices.

## 4 Conclusions

This study revealed that the type and concentration of milk proteins (CS or WPI) incorporated into hydrogels significantly influence the structural and mechanical characteristics of BGs. BGs containing CS exhibited the highest firmness at 1 % protein concentration but showed phase inversion at higher levels. In contrast, WPI-based BGs consistently maintained a hydrogel-in-oleogel structure. Compared to WPI–KGM mixtures, CS–KGM mixtures formed more robust interfacial networks, resulting in reduced oil-water interfacial tension and distinct textural behavior. These results highlight the potential of tailoring BG properties through protein selection, offering promising applications as alternatives to conventional solid fats. Further studies will focus on evaluating their sensory performance and long-term storage stability.

## CRediT authorship contribution statement

**Lijuan Han:** Writing – review & editing, Supervision, Project administration, Funding acquisition, Conceptualization. **Shiqi Zhang:** Writing – original draft, Investigation. **Yingxin Chen:** Software, Methodology. **Lingzhi Su:** Software, Methodology. **Junbo He:** Resources, Formal analysis. **Weinong Zhang:** Supervision, Conceptualization.

## Declaration of competing interest

The authors declare that they have no known competing financial interests or personal relationships that could have appeared to influence the work reported in this paper.

## Data Availability

No data was used for the research described in the article.

## Appendix A Supplementary data

Supplementary material. Image 1
