# Supplementary figures and images for: Comparison of the physical properties and microstructure of bigels prepared from ethyl cellulose oleogels and konjac glucomannan hydrogels containing casein or whey protein isolate
Source: Food Chem X. 2025 Aug 5;29:102880. doi: 10.1016/j.fochx.2025.102880 (PMC12355583; doi:10.1016/j.fochx.2025.102880)

**Appendix A:**

**Supplementary material**


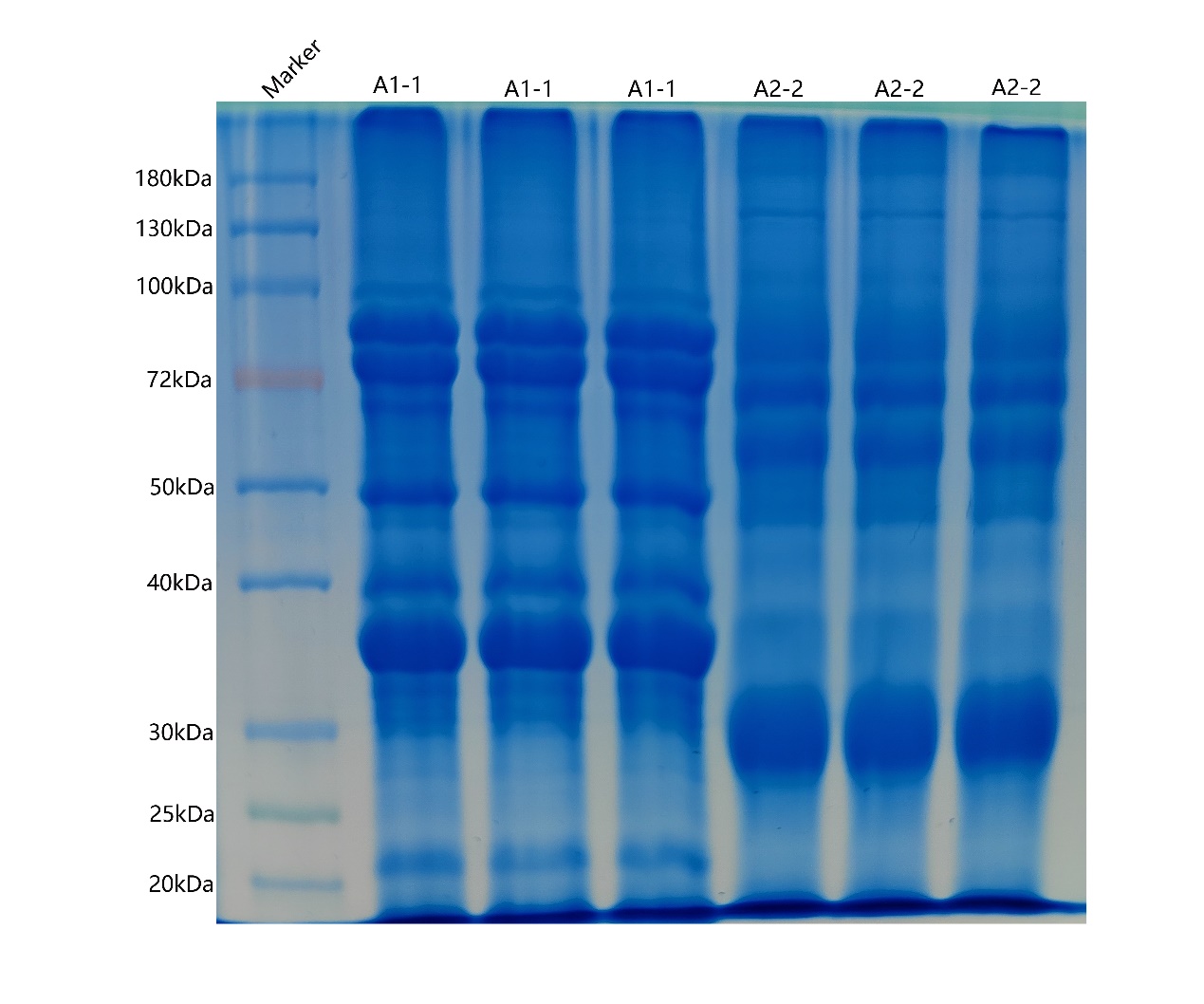


Figure S1. SDS-PAGE profiles of CS (A1) and WPI (A2).

Supplement: Supplementary file 1 — Supplementary material. [file mmc1.docx]
